# Genomic-regions associated with cold stress tolerance in Asia-adapted tropical maize germplasm

**DOI:** 10.1038/s41598-023-33250-8

**Published:** 2023-04-18

**Authors:** Kumari Shikha, Vinayan Madhumal Thayil, J. P. Shahi, P. H. Zaidi, Kaliyamoorthy Seetharam, Sudha K Nair, Raju Singh, Garg Tosh, Ashok Singamsetti, Saurabh Singh, B. Sinha

**Affiliations:** 1 Department of Genetics and Plant Breeding, Banaras Hindu University (BHU), Varanasi, India; 2 International Maize and Wheat Improvement Centre (CIMMYT), ICRISAT Campus, Patancheru, Telangana India; 3 Borlaug Institute for South Asia (BISA), Ludhiana, Punjab India; 4 Punjab Agricultural University (PAU), Ludhiana, India

**Keywords:** Plant breeding, Agricultural genetics

## Abstract

Maize is gaining impetus in non-traditional and non-conventional seasons such as off-season, primarily due to higher demand and economic returns. Maize varieties directed for growing in the winter season of South Asia must have cold resilience as an important trait due to the low prevailing temperatures and frequent cold snaps observed during this season in most parts of the lowland tropics of Asia. The current study involved screening of a panel of advanced tropically adapted maize lines to cold stress during vegetative and flowering stage under field conditions. A suite of significant genomic loci (28) associated with grain yield along and agronomic traits such as flowering (15) and plant height (6) under cold stress environments. The haplotype regression revealed 6 significant haplotype blocks for grain yield under cold stress across the test environments. Haplotype blocks particularly on chromosomes 5 (bin5.07), 6 (bin6.02), and 9 (9.03) co-located to regions/bins that have been identified to contain candidate genes involved in membrane transport system that would provide essential tolerance to the plant. The regions on chromosome 1 (bin1.04), 2 (bin 2.07), 3 (bin 3.05–3.06), 5 (bin5.03), 8 (bin8.05–8.06) also harboured significant SNPs for the other agronomic traits. In addition, the study also looked at the plausibility of identifying tropically adapted maize lines from the working germplasm with cold resilience across growth stages and identified four lines that could be used as breeding starts in the tropical maize breeding pipelines.

## Introduction

Maize acreage has been increasing due to its higher demand and profitability owing to its diverse uses such as feed, food, fuel, and industrial raw material^1^.In Asian tropics, maize is largely grown as a rainfed crop in the rainy season^2^. Over the recent years, winter season maize is gaining importance in areas with assured irrigation facilities, due to higher yield potential and low incidence of pest and diseases. However, maize cultivation during winter season particularly in Asian tropics is constrained when the seasons temperatures drops below 10 °C. Temperatures below 20 °C is known to restrict growth and development of maize^3^. Sub-optimal temperature in crops affects the growth from delayed emergence to poor seed-setting^4,5^ and could lead to a complete loss of crop yield. Low temperatures during early seeding stages diminishes leaf development^6–8^, while the stress at vegetative stage often decreases plant height and causes chlorosis and reduces photosynthetic efficiency^9–12^ and the losses. At reproductive stage the stress delays anthesis, increases anthesis silking interval (ASI), decreases pollen shed and eventually leads low seed set^13^. Use of cold tolerant hybrids along with suitable agronomic practices^14,15^ might help mitigate the adversities of low temperatures^16,17^.

One of the major challenges in breeding for cold stress is availability of a reliable high throughput screening facility for imposing the stress on large-scale screening materials. Screening of large number of germplasm particularly in field would entail adjusting the time of planting, such that the targeted crop stage is exposed to the stress^18,19^. However, it is often difficult to predict with accuracy and duration of the low temperature regime that matches the targeted crop stage(s) ^20^. Using growth chambers for smaller studies might be a feasible option, but such conditions may not be suitable for large-scale screening of germplasm essential to any breeding program particularly due to the intensive cost and labour. Further, the complexity of the stress in addition to the uncertain relationship between these artificial and natural screens and varied target population of environments (TPEs) could often hamper in obtaining tangible outputs of the pipeline^18,21,22^.

In addition, most of these studies were limited to improving cold tolerance at germination and seedling stage^23,24^ and/or were largely based on maize germplasm adapted to temperate climates. It is comparatively easier to mitigate the germination/ seedling stage cold stress through alterations to planting dates^25,26^, however the uncertainty of the onset of cold waves/ cold snaps during winter season at later stages of crop growth (Vegetative or flowering) necessitates the use of germplasm that show cold tolerance at these key growth stages.

Complexity of the cold tolerant traits along with high genotype × environment interaction suggests the use of molecular markers in breeding for cold stress tolerance^27^. Molecular markers would largely assist breeders in developing materials with reasonable level of cold tolerance that are suitable for winter season cultivations by circumventing the need for large scale evaluations of germplasm in breeding pipeline. Earliest report of QTL mapping study on maize seedling tolerance to cold stress was by Frashboud et al.^28^ on a set of recombinant inbred lines. QTLs for cold stress at seedling stage have since been identified in several studies ^27–36^. A meta QTL analysis^34^ involving seven studies identified major regions on chromosomes 2,4 and 8 regulating early development of maize seedlings under cold conditions. The differences in numbers, effects, and positions of QTL in various studies indicate that cold stress tolerance is a very complex trait and is dependent on the population, environment and the stage of the crop that is exposed to the stress. Leipner et al.^27^ identified QTLs for flowering time, plant height and biomass at harvest on the mapping population used in a previous study screened under suboptimal conditions in field. The study, however, did not co-map any major QTL that was associated with early seedling stage chilling tolerance to the later stage traits, suggesting that maize has higher compensation capacity and the conditions during later stage of crop may be more important in determining the final biomass accumulation. Further, studies involving transcriptome analysis have also been done to understand the molecular basis of the cold stress in maize^26,37–39^.

Considering the limitations and low resolution of QTLs detected through linkage mapping, Genome wide association study (GWAS) that exploits historical recombination was conducted on a set of North American and European dent and European Flint lines exposed to cold stress during seedling stage^21^. The study revealed three important genomic regions for photosynthetic capacity traits for seedling stage cold tolerance on chromosomes 1, 5 and 7. A similar study on two major maize panels^40^ identified few major genomic regions (Chromosomes 1,3,4,5 and 7) for photosynthetic capacity and early vigor under cold stress. A study on early-stage cold tolerance revealed SNPs on chromosomes 1, 2, 4 and 7 for germination related traits^41^. Similar study on a panel of 283 maize lines was conducted by Hu^42^ and identified 17 SNPs associated with germination traits under cold stress environments. Zhang et al.^43^ also reported several genomic regions for fourteen germination traits associated with cold tolerance on a panel of 222 maize inbred lines. A recent GWAS on 406 inbred lines^44^ derived from multiparent advanced generations inter-cross (MAGIC) population revealed the significance of chromosome 10 (bin10.04) as hot spot for photosynthetic traits associated with early-stage cold stress in maize. The most recent study by Yi et al.^17^ on a panel of 836 diverse maize lines revealed close to 159 loci associated with cold stress related germination and seedling traits, few of the QTLs identified in this study co-mapped to previous QTL mapping studies. Although several genomic regions have been identified for early crop stage cold tolerance in temperate adapted maize, little to no information is available on vegetative/ flowering stage cold stress tolerance particularly for cold snaps^45^ that are frequented in winter season maize in tropical maize. In the current study, the test crosses from a panel of 297 Asia adapted tropical maize lines were evaluated for cold stress tolerance at early various growth stages. The study envisaged i) identifying putative regions for cold tolerance associated traits such as grain yield, flowering and plant height across different stages of crop growth in this working germplasm and ii) comparing these results to regions previously identified for the traits associated with early-stage cold stress tolerance.

## Results

### Trait and genotypic variability

**Figure 1:**
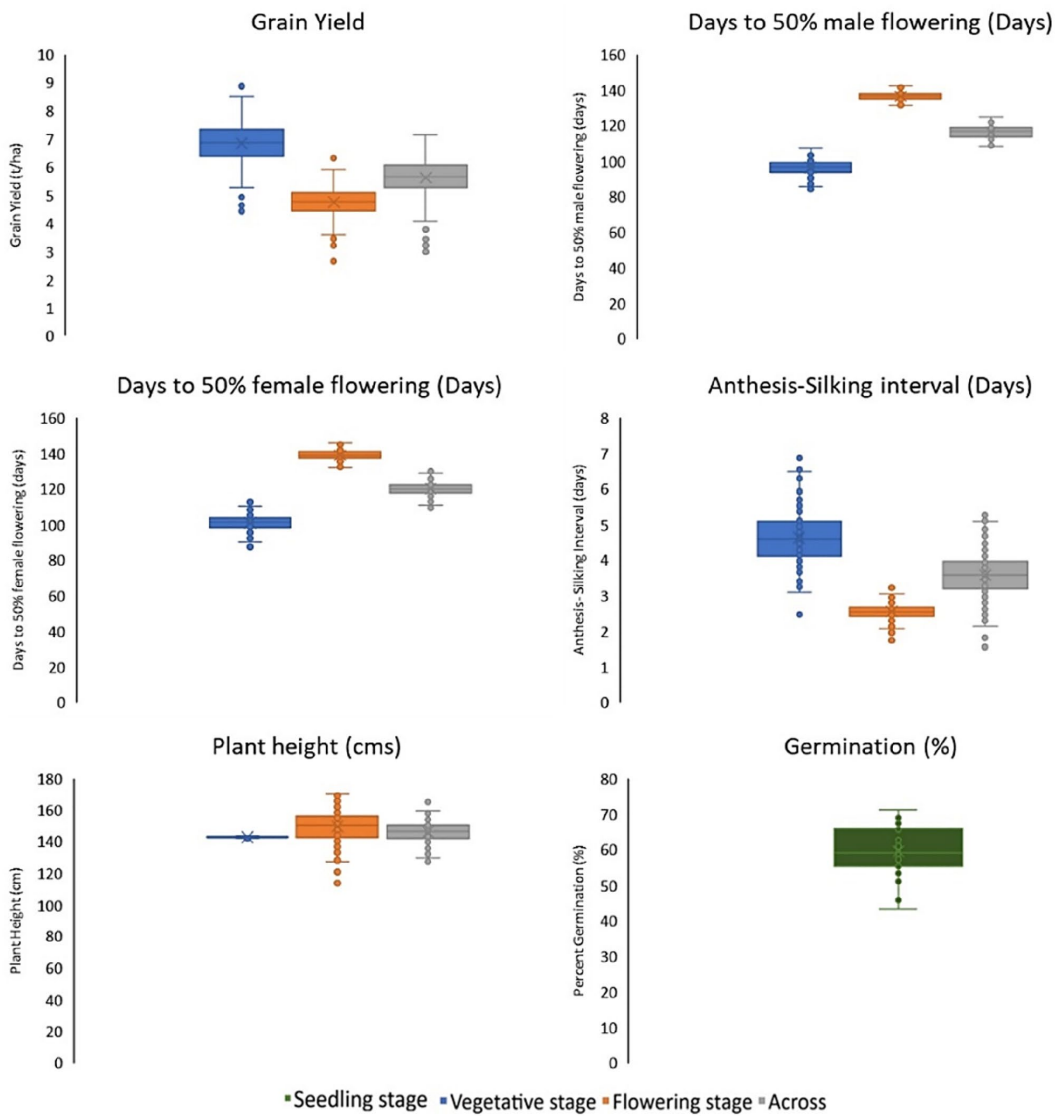
Performance of entries across vegetative, flowering and across season datasets.

**Figure 2:**
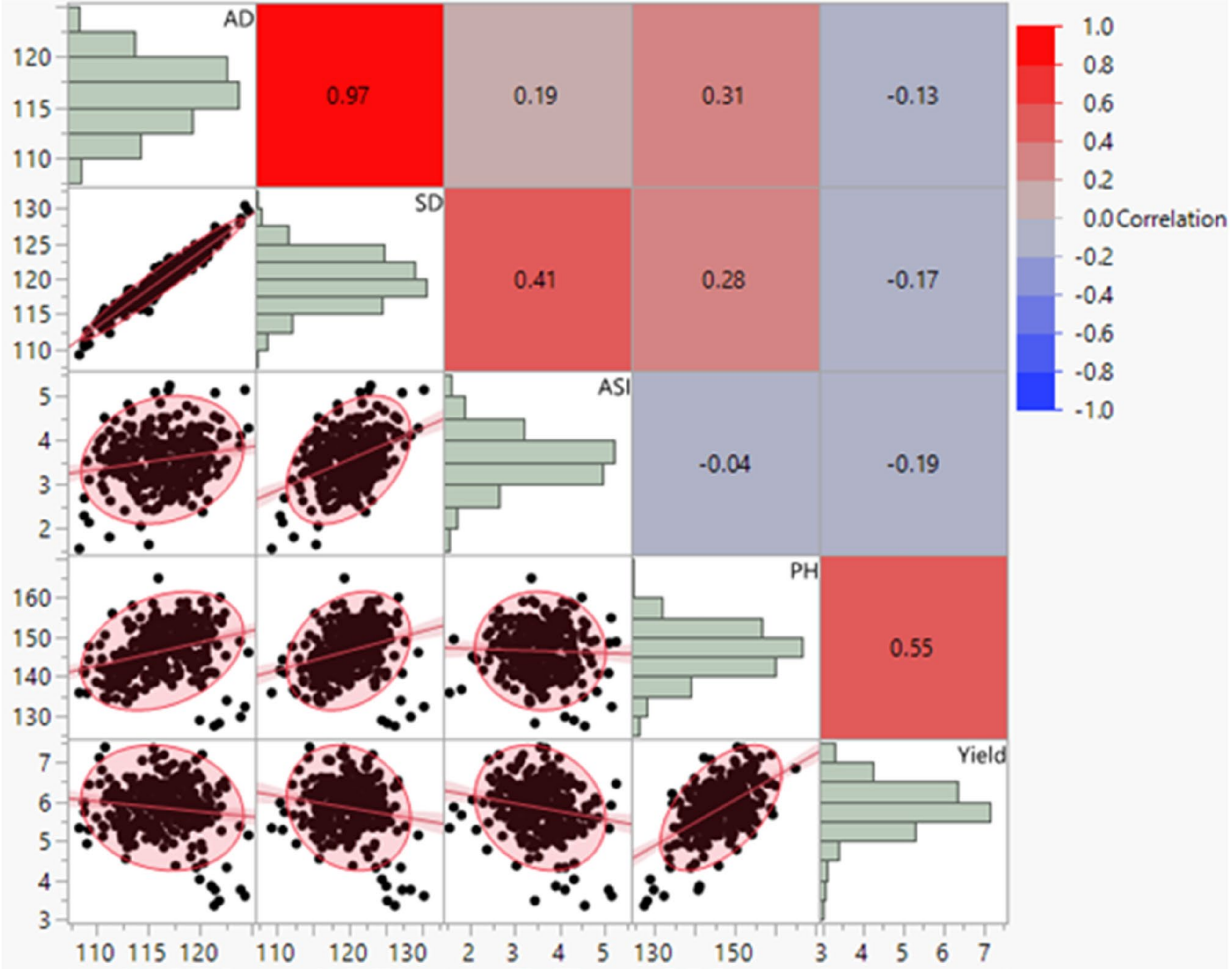
Scatter plot, frequency distribution and correlations coefficients among the traits recorded across cold stress locations (L2 to L5).

Significant effects of cold stress were observed among the entries exposed to cold stress at different growth stages across the test locations (Table1). Substantial genotypic variation (> 70%) was recorded amongst the entries for flowering date followed by the trait plant height and grain weight (Fig. 1 and Table 2). Cold stress prolonged the vegetative state of the planted crop and invariably extended flowering time of the crop (> 90 days). The most substantial effect of cold stress environments could be observed in the trial planted at L4 (Ludhiana_2019), where the average days to 50 percent flowering in the entries was 160 days followed by L5 (Varanasi_2019). The stress affected both male and female flowering in the crop and as expected the variation was highest in L4, where the trials were exposed to cold stress and remained suspended at vegetative stage for a very long duration. The mean grain yield in the trials at L2 and L3 (> 6.0 t/ha) was higher than that observed for in L4 and L5 (~ 5 t/ha). Similarly, the average plant height at this location (L4) was high, with large number of entries planted reaching above 150 cm. However, the heritability for the trait in L4 was low (0.24). Most decipherable effect of cold stress in field condition was recorded on the plant stand in L1 (Ludhiana_2018). The trial at this location was planted in sub optimal temperatures to study the effect of low temperatures on the germination ability of the entries (58 percent). Agronomic traits such as days to 50 percent flowering, plant height or yield were not recorded in this trial due to the poor plant stand. Among the entries four most stable and adaptable entries were identified based on stability parameters and their performance across the cold stress tolerance at different crop stages are presented in Table 3. The coefficient of regression recorded for the entries along with standard deviation are presented in supplementary Table 1. Most of the traits observed under cold stress showed near normal distribution across locations with a slight skew for plant height which showed strong association with grain yield (R^2^ = 0.50). The association of the flowering traits among themselves was positive and significant (R^2^ > 0.98) while this relationship was negative and weak with grain yield (R^2^ < − 0.20) (Fig. 2).

**Table 1:** Location and date of planting and crop stage exposed to low temperature.

Environment and year	Longitude and latitude	Location	Date of planting	Stage of crop plant exposed to cold stress
Ludhiana_2018	30.90°N, 75.80°E	L1	11-Dec-18	Seedling
Samastipur_2018	25.95°N, 85.66°E	L2	10-Oct-18	V7 to VT
Varanasi_2018	25.26°N, 82.99°E	L3	18-Oct-18	V7 to VT
Ludhiana_2019	30.99°N, 75.74°E	L4	7-Nov-19	V3 to V6
Varanasi_2019	25.26°N, 82.99°E	L5	10-Nov-19	V3 to V6

**Table 2:** Descriptive statistics of test crosses of panel of tropical Asia adapted lines under cold screens.

Statistic	Location	Yield (t/ha)	AD (days)	SD (days)	ASI (days)	PH (cm)	Germ (%)
Mean	L1						58.52
	L2	7.74	93	98	5	136.58	
	L4	5.34	161	161	1	187.06	
	L3	6.04	99	104	5	149.19	
	L5	4.19	112	116	4	112.96	
	Across	5.84	116	120	4	146.47	
LSD	L1						22.58
	L2	2.35	5	5	2	12.46	
	L4	2.31	2	3	1	15.41	
	L3	2.09	5	6	2	13.31	
	L5	1.13	3	3	2	13.22	
	Across	1.31	3	3	1	12.09	
Genotype Var	L1						121.77
	L2	2.01**	17.59**	20.88**	1.07**	26.36*	
	L4	0.91**	4.58**	6.72**	0.36**	150.89**	
	L3	1.46**	20.13**	22.97**	2.05**	146.83**	
	L5	0.62**	15.47**	15.64**	1.01**	163.48**	
	Across	0.65**	11.64**	13.70**	0.55**	57.62**	
Gen × Loc Var	Across	0.62**	2.85**	2.95**	0.58**	65.09**	
Env. Var Heritability	Across	2.09*	929.19	822.27	3.39	940.91*	
	L1						0.47
	L2	0.68	0.87	0.87	0.66	0.24	
	L4	0.61	0.87	0.88	0.40	0.85	
	L3	0.63	0.85	0.85	0.73	0.90	
	L5	0.77	0.94	0.93	0.66	0.89	
	Across	0.67	0.91	0.91	0.65	0.69	
Residual Var	L1						273.96
	L2	1.89	5.27	6.35	1.11	165.13	
	L4	1.18	1.35	1.91	1.10	53.86	
	L3	1.72	6.95	7.92	1.51	33.37	
	L5	0.38	2.04	2.31	1.04	42.21	
	Across	1.28	3.97	4.64	1.19	76.71	

*,**Represents significance at P ≤ 0.01 and ≤ 0.001 respectively.

AD, SD, ASI and Germ represents Days to 50% anthesis, Days to 50% silking, Anthesis-silking interval and Germination respectively.

**Table 3:** Test cross performance of selected Asia adapted lines under cold stress across different crop growth stages.

Entry	Source of pedigree	Management/stage of cold stress	AD (days)	SD (days)	PH (cm)	ASI (days)	Yield (t/ha)
CIMIND-425	Tropical yellow QPM pop	Across	116	120	152.5	4	6.1
		Flowering	96	100	95.5	4	7.5
		Vegetative	137	140	161.1	3	4.4
DTMA-100	Multiple insect resistant pop. 590	Across	118	122	153.3	4	6.9
		Flowering	99	104	99.2	5	7.9
		Vegetative	138	140	161.7	3	5.2
DTMA-170	Subtropical Intermediate Yellow Dent pop. 45	Across	115	117	150.1	3	5.9
		Flowering	95	98	94.6	4	7.2
		Vegetative	135	136	151.3	2	3.7
DTMA-194	Synthetic yellow Tar spot resistant pop. and Tropical yellow Dent pop. 24	Across	119	123	152.2	4	6.2
		Flowering	99	104	99.4	5	7.7
		Vegetative	139	141	151.1	2	4.2

AD, SD and ASI represents Days to 50% anthesis, Days to 50% Silking and Anthesis-silking interval respectively.

### Population structure and Linkage disequilibrium

**Figure 3:**
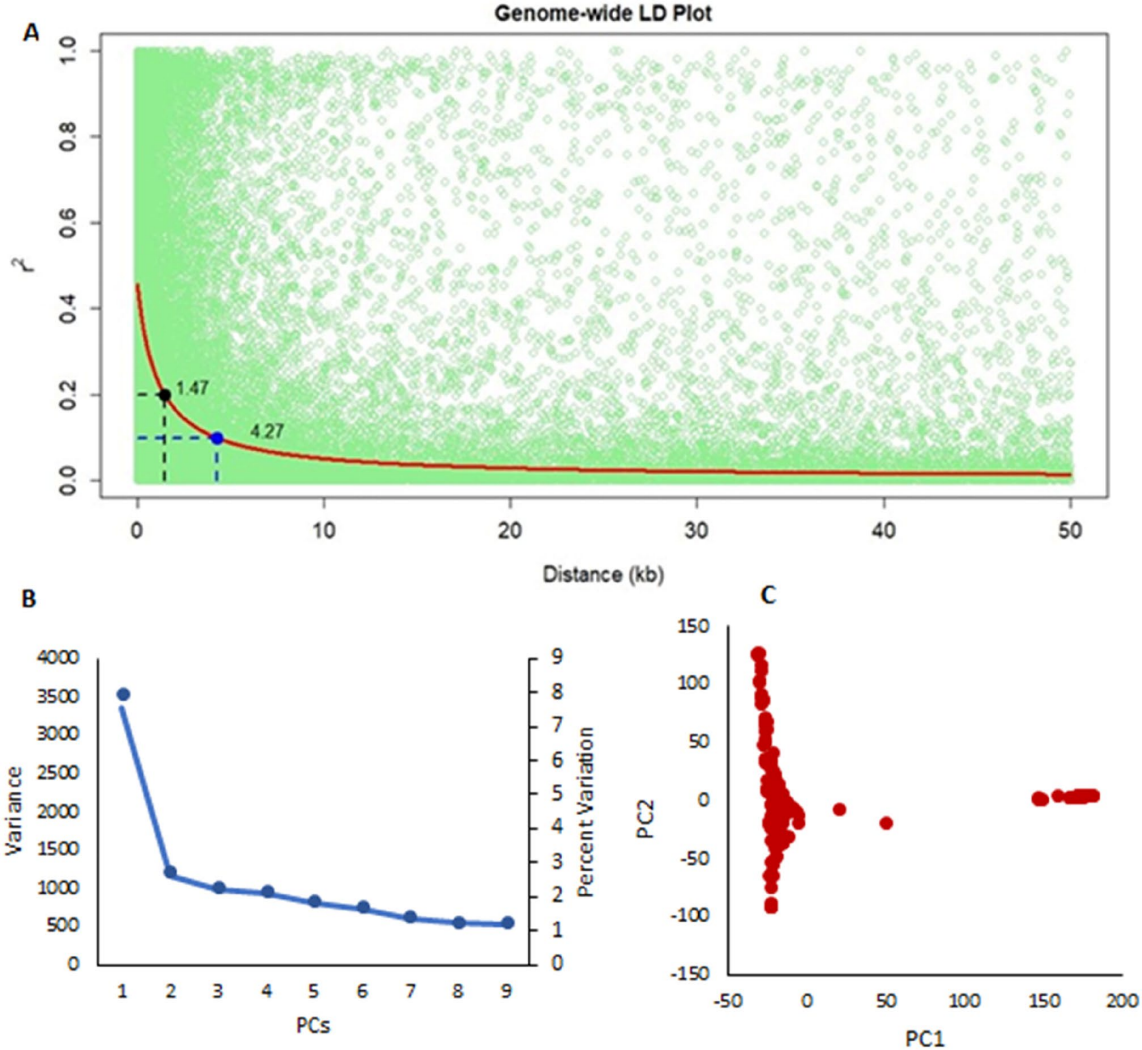
LD-decay (**A**), scatter plots of 10 PCs (**B**) and population structure as expressed by first two PCs (**C**) observed among the panel of Asia-adapted tropical lines.

The population showed a high LD decay of 1.5 Kb at r2 of 0.2 and 4.27 Kb at r2 of 0.1 (Fig. 3). SNP associations identified were corrected for structure using three principal components cumulatively accounting for close to 15% of the variation (Supplementary Material Fig. 1). GWAS analysis was done on the primary trait grain yield under cold stress at the four test locations using the multi-locus models (MLM, mrMLM, MLMM, BLINK and Farm CPU). Significantly associated SNPs were identified and compiled through multi locus model “BLINK” as it had the best fit Q–Q plots (Supplementary Material Fig. 2a and 2b with least amount of genomic inflation for the primary trait- Grain yield across the environments. Considering that the test environments in this study were varying with significantly high environmental variance, the GWAS analysis was done on three combined datasets involving vegetative stage cold stress environments (L4 and L5), flowering stage cold stress environments (L2 and L3) and across all environments. The results were then compared to identify common significant SNPs across these datasets. For the study we used a threshold of P < 1 × 10^−5^, against a holm and bonferroni *P* value (< 0.01) which was too stringent in identifying top SNP association across the different environment data sets.

### Association mapping

Large number of significant SNP associations at *P* value P < 1 × 10^−5^ were identified for the traits studied across the different environments (Supplementary Material Table 2, Suppl. Figure 3), however, several of the most significant associations (*P* value < 0.01) across the traits and locations congregated on chromosomes 3 (bin 3.05–3.06) (Table 4). Among the traits studied, most significant SNPs for percent germination were found on chromosomes 4 (bin 4.08) and 8 (bin8.03). The SNP on chromosome 8 (S8_21830966) recorded the highest allelic effect (> 3% germination). Significant SNPs for flowering traits (AD, ASI and SD) were also found on most of the chromosomes with most significant SNPs accounting for more than 10% percent of phenotypic variation in vegetative stage cold stress environments on chromosomes 1 (bin 1.04), 3 (bin 3.06), 7 (7.02) and 8 (8.06) and on chromosomes 1 (bin1.02, 1.09), 5 (5.06) and 9 (bin9.07) for flowering stage cold stress locations. The SNPs identified for male flowering on chromosomes 5 (bin 5.06) and 8 (bin8.06) co-localised with the identified significant SNPs for female flowering accounting for more than 10 percent of the phenotypic variation across the two datasets.

**Table 4:** Chromosomal regions with most significant SNPs (Bonferroni and holm *P* value < 0.01) traits associated with cold tolerance.

Trait	Stage of cold stress	SNP	Chr	Bin	Gene model	Pos. (Mb)	*P* value	maf	effect	Phenotypic variance (%)	Associated protein/enzymes
Grain Yield	Vegetative	S2_14539348	2	2.02	GRMZM2G314396	15	3.7E−08	0.09	0.27	37.02	*Calcium dependent protein Kinase5*
		S2_45988012	2	2.04	GRMZM2G028258	46	1.5 E−08	0.21	− 0.22	38.13	Potassium channel SKOR
		S3_169264156	3	3.06		169	1.8 E−07	0.12	− 0.24	6.21	
	Flowering	S1_286656232	1	1.11		287	1.1 E−09	0.20	0.19	8.39	
		S2_213163019	2	2.08	GRMZM2G703373, GRMZM5G895355	213	7.0 E−10	0.06	0.37	24.74	Pentatricopeptide repeat-containing protein
		S3_143356181	3	3.05	GRMZM2G053343	143	9.3 E−11	0.19	0.24	11.10	Elongation factor G
		S7_162341622	7	7.04		162	5.7 E−09	0.11	0.23	11.15	
Days to 50% anthesis	Vegetative	S2_196110540	2	2.07		196	1.7 E−09	0.49	− 0.88	7.88	
		S3_185976288	3	3.06	GRMZM2G021881	186	1.2 E−07	0.10	1.29	10.56	Calcium uniporter protein
		S4_158607550	4	4.06	GRMZM2G106795	159	6.9 E−08	0.19	0.87	5.77	ADP-ribosylation factor B1B
		S7_104175420	7	7.02	GRMZM2G032160	104	1.8 E−07	0.07	− 1.32	21.56	*endo-1,3(4)-beta-glucanase*
		S8_149609962	8	8.06		150	3.4 E−08	0.09	1.25	8.62	
		S9_90843159	9	9.03	GRMZM2G056093	91	3.0 E−08	0.44	0.78	6.52	*Tetratricopeptide repeat (TPR)-like superfamily protein*
	Flowering	S1_21543873	1	1.02		22	2.1 E−07	0.35	0.48	12.52	
		S1_250912433	1	1.09		251	4.0 E−11	0.13	− 1.07	28.18	
		S5_196412134	5	5.06		196	2.4 E−09	0.25	0.68	14.82	
	Across	S3_186214261	3	3.06		186	3.6 E−09	0.10	1.06	48.04	
		S9_90843159	9	9.03	GRMZM2G056093	91	1.6 E−09	0.45	0.57	17.32	*Tetratricopeptide repeat (TPR)-like superfamily protein*
Days to 50% Silking	Vegetative	S1_53467993	1	1.04		53	1.6 E−07	0.34	0.87	15.19	
		S8_149609962	8	8.06		150	1.0 E−09	0.09	1.63	28.27	
	Flowering	S5_196412120	5	5.06		196	3.2 E−11	0.33	0.81	33.65	
		S9_147965995	9	9.07		148	9.1 E−08	0.35	− 0.64	22.12	
Plant height (cm)	Flowering	S3_153769448	3	3.05		154	5.3 E−08	0.09	− 3.62	64.05	
		S6_93497825	6	6.02	GRMZM2G161680	93	7.0 E−08	0.50	1.91	11.98	CCAAT-HAP5 transcription factor
	Across	S3_152451938	3	3.05	GRMZM2G170689	152	6.3 E−08	0.07	3.06	17.34	*S-formylglutathione hydrolase*
		S3_153769448	3	3.05		154	1.1 E−09	0.09	− 2.93	17.78	
		S5_206305411	5	5.07	GRMZM2G039855	206	4.3 E−10	0.06	− 3.46	31.46	
		S8_72384070	8	8.03	GRMZM2G063880	72	5.9 E−08	0.35	1.56	6.69	WRKY-transcription factor 106

**Figure 4:**
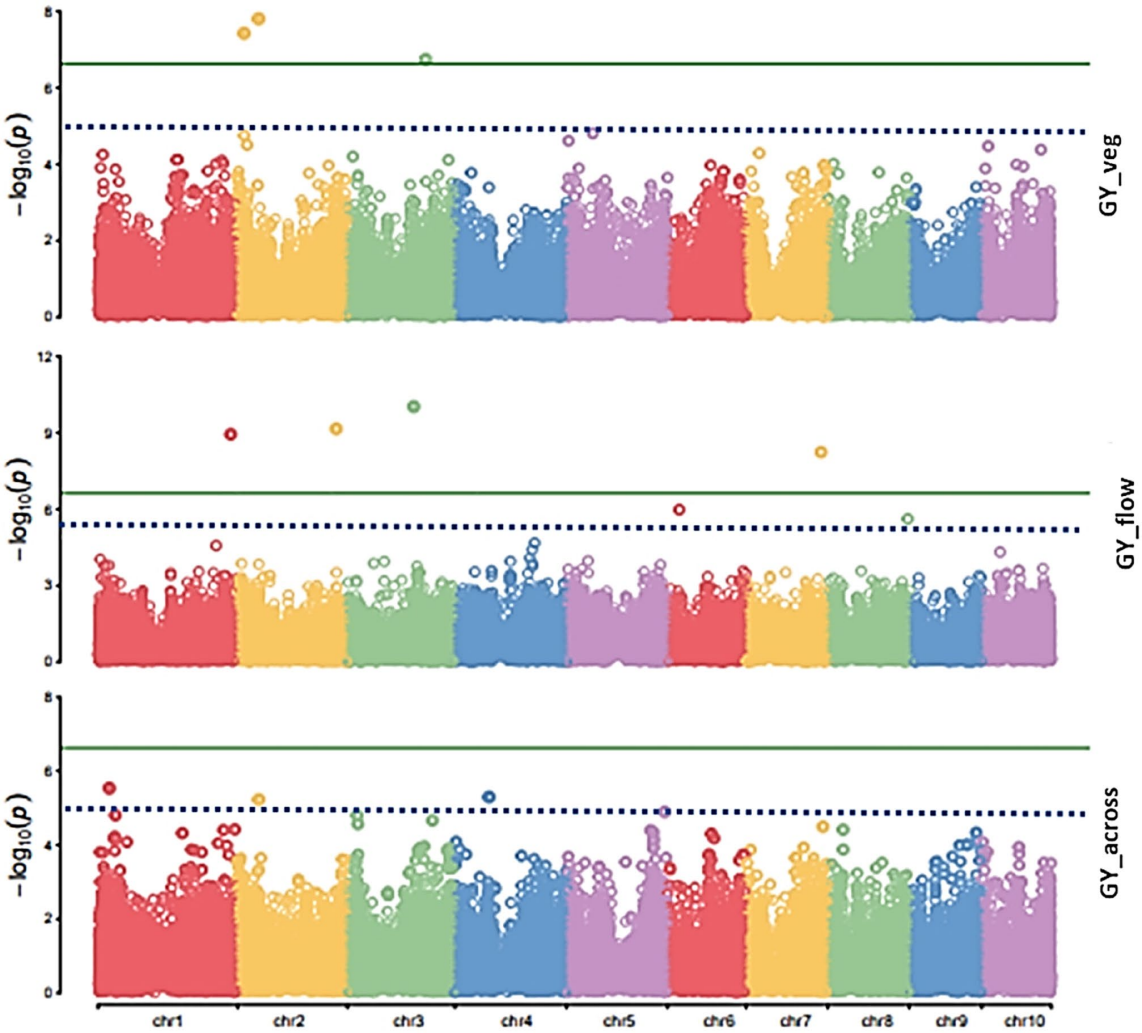
Manhattan plot representing the regions significantly associated with grain yield under vegetative (veg), flowering (flow) and across (across) location datasets.

Significant SNPs for plant height were found distributed across the chromosomes for flowering and across environment datasets. The SNP on chromosome 3 (S3_153769448) and 5 (S5_206305411) accounted for more than 15% of the phenotypic variation. Significant SNPs for the trait grain yield (adjusted for plant height) were also found distributed across most of the chromosomes, however the SNPs on bins 2.02 (S2_14539348) and 2.04 (S2_45988012) for flowering stage cold stress datasets and bin 2.07 (S2_213163019) for vegetative stage cold stress are of particular interest as they accounted for more than 20% of the phenotypic variation (Table 4, Fig. 4). The complexity of the trait grain yield is evident as the study did not identify any common SNPs across the datasets. Hot spots with significant QTNs (*P* value < 0.5 × 10^–5^) for multiple traits were also observed in the study (Table5). The region on bin 3.05–3.06 and 8.05–8.06 are of particular interest, as QTNs of multiple traits congregated to these region.

**Table 5:** Chromosomal regions with significant SNPs (*P* value < 0.5 × 10^–5^) associated with multiple traits across locations and their potential gene functions.

Bin	Chromosomal position (Mb)	Associated Traits & Dataset	Putative gene function with significant marker trait association
1.04	53–55	PH_Ac,SD_Ac,SD_Veg,SD_flow	*Acid phosphatase*- Phosphate acquisition
2.07	196.11	AD_Ac,AD_Veg, SD_Veg	
3.02	5.84	PH_Ac,SD_Ac	*Serine/threonine-protein kinase* *ALE2*
3.05	142–152	AD_flow,PH_flow,PH_veg, GY_flow	*S-formylglutathione hydrolase*
3.06	185–195	AD_Veg, AD_Ac	*Calcium uniporter protein, Peptide transporter PTR2*
4.09	226–236	PH_Ac, SD_Ac, SD_flow	*kch3—potassium channel3*
5.06–5.07	196–206	SD_Flow, AD_Flow, PH_flow	*kinesin-like protein KIN-4C*
8.05–8.06	145–150	PH_Ac, SD_Ac, AD_Veg, SD_Veg, AD_Ac	*Signal recognition particle 54 kDa protein*
8.08	172–173	PH_flow, FW_flow	*Glutathione transferase*
9.03	90.84	AD_Ac, AD_Veg	*Tetratricopeptide repeat (TPR)-like superfamily protein*
9.07	147–148	AD_flow,AD_Ac, SD_flow	*40S ribosomal protein S3a*

GER, PH, GY, AD, SD, ASI represents percent germination; plant height, grain yield, days to 50% anthesis, days to 50% Silking and Anthesis-silking interval respectively and veg, flow and Ac represents the vegetative stage, flowering stage and across location dataset.

### Haplotype regression

A subset of SNPs was selected to perform a haplotype trend regression on the grain yield dataset of the current study. A set of 292 SNPs with *P* value ≤ 1.0 × 10^−3^ in the association tests for grain yield across location dataset formed 50 haplotype blocks. The regression analysis identified 6 of these haplotype blocks with Bonferroni *p* value of less than ≤ 0.05 (Table 6). These blocks were found congregated on chromosomes 2, 5, 6 and 9. The highest phenotypic variation was accounted for by the blocks on chromosome 5 (bin 5.07 and bin 5.05) followed by on chromosome2 (bin2.02).

**Table 6:** Significant haplotypes identified for grain yield across environments.

Block	Bin	Markers in the block	Gene model	No. haplotypes	Phenotypic variance (%)	Putative gene function/association
22	5.07	S5_204753139, S5_204753169	GRMZM2G341479	2	9.63	*Pentatricopeptide* repeat61
21	5.05	S5_194045435, S5_194045477	GRMZM2G106185	2	8.80	*G2-like-transcription factor 20 (glk20)*
8	2.02	S2_9099519, S2_9099551	GRMZM2G000047	2	6.38	*hak*28—potassium high-affinity transporter28
35	9.03	S9_74588195, S9_74588196, S9_74588198, S9_74588200	GRMZM2G366146	2	6.23	ABC transporter G family member 29
23	6.02	S6_89687259, S6_89687831, S6_89825614	GRMZM2G044398, GRMZM5G804555, GRMZM2G082322	2	5.54	Zinc finger CCCH domain-containing protein 40
24	6.02	S6_89825705, S6_89825727	GRMZM2G082322	2	4.83	CID domain-containing protein

## Discussion

Cold stress is one of the major abiotic stresses encountered by tropical maize cultivated during off-season (Winter season) particularly in Asian tropics, causing irreversible physiological damages and severe yield losses^26,46–52^. The unpredictability of cold snaps during the winter season is a major impediment to adoption of maize for cultivation. Consequently, it is pertinent to have germplasm resilient to cold stress particularly for products that are targeted to this season.

Variability for cold stress tolerance within tropical germplasm has been reported in previous studies^19^. The current study also showed substantial variation for this stress except for the trait germination percentage with most entries in L1 showing poor plant stand. The poor germination percentage (< 50 percent) in the trials sown in December at L1 is attributed to the low temperatures prevailing during the planting of the trial. The effect of low temperatures on germination in maize has been extensively reviewed in several studies^14,53,54^. The poor germination rates observed in the current study however hindered reliable estimation of other morpho-physiological traits such as grain yield plant height or days to 50 percent flowering, and this trial was limited to studying the effect of cold stress on seedling germination. In addition, cold tolerance to germination is unlikely a breeding target for off season planting in tropical environments, as this stress can also be mitigated largely through changes in planting time^14^. Apart from germination, the most evident effect of cold stress was observed on the trait days to 50% flowering. In both the testing years, the flowering stage of the trials were delayed. However, the earliest flowering (< 100 days) was recorded in trials planted during 2018 (L2 and L3), wherein the vegetative stage (V1 to V5) of the crop was not exposed to low temperatures and sub-optimal temperatures were primarily reported during V9 to VT stage of the crop. Cold stress delays flowering of most of the crops^55^, however, sub-optimal temperatures just before or at the initiation of floral transition is reported to inhibit branch meristem development and number of spikelet pairs^56^ in maize. These locations, however reported comparatively higher grain yields than L4 and L5 where the crop was exposed to cold stress during vegetative stage. The entries in these trials planted at L4 and L5 remained suspended in vegetative state (V3 to V6) for longer duration further delaying the flowering till the temperatures improved. Interestingly, the flowering initiation in the entries were slow initially due to cold stress and hastened particularly in L4 as the temperature improved during later part of the crop cycle. All the entries in this location (L4) flowered within an interval of 10 days as against more than 20 days observed in other trials. Zaidi et al.^19^ suggested that the genotypes which required more days to complete their vegetative growth had fewer days for reproductive growth, resulting in lower per day yield, and eventually yielded very low.

Cold stress also impedes the cell cycle and rate of cell division^57^ there by slowing the growth rate of crops. The rate and duration of seed filling are also reported to decrease with prolonged exposure to cold stress at early grain filling^58^. Even short spells of cold nearing 5 °C at the V3–V5 growth stages are reported to cause photoinhibition^51^, chlorosis, membrane damage, and eventually necrosis^59^ or even plant death^52^. As expected, reduced plant height was observed in most of the test locations where the crop was exposed to cold stress, however this effect was not prominent in L4 as the entries were taller in this location. This difference might possibly be a result of elongated internodes due to poor sun-light intensity at this location during winter season. Severe cold stress in general is reported to reduce the plant height along with total crop biomass in maize^19,60^. However, it is reported that moderate temperatures for few days (temperatures 10–12 °C) preceding severe cold stress slows the growth and development of the crop but does not cause permanent injury^26,61^. In L4, the initial daily temperatures (Tmin) were observed to 10–12 °C for nearly a month prior to prolonged severe cold stress possibly contributing to cold acclimation of the crop. Plant height has been suggested in few previous studies as one of the primary candidates for breeding for stability due to its non-relevant interactions with environments and positive effects on yield^62^. Considering the high variation observed among the different environments, the performance of entries particularly for grain yield varied. The identification of only four stable and adaptable entries out of 300 entries screened across environments, suggests that resilience do exist, though at very low frequency in the tropical germplasm for cold stress. Expanding the trial size and accommodating more germplasm in screens may result in identifying more of stable germplasm for tolerance to cold stress. The test crosses from these lines performed reasonably well in all the cold stressed environments and had showed reasonable levels of gemination percentages. However, considering these lines are not elite, they would be most suitable as donor lines or sources of enriching favourable alleles in populations that exist for developing elite cold resilient inbred lines in the program.

Mapping of QTLs in biparental families and genome wide association studies on diversity panels are the two most used approaches in identifying genomic regions associated with the trait of interest. GWAS approach uses historical recombination events and larger allelic diversity compared to family-based mapping approach to identify potential trait -marker associations. In the current study, we used GWAS approach to identify SNP markers associated with cold stress tolerance in a panel of tropical maize lines screened under field conditions. The current panel of lines had a high LD decay which is typical of tropical germplasm^63–65^, which indicates the diversity among the lines involved and the amenability to high resolution mapping studies.

The current study identified a suite of significant SNPs for various traits recorded under cold stress at different crop stages across various locations. Poor germination in seedling stage exposed to cold stress is one of the major traits that could adversely affect the final plant stand in the field there by impacting the realised yield. In the current study, SNPs have been identified for this trait in most of the chromosomes The SNP with the lowest *P* value (4.32 × 10^–6^) was observed on chromosome 4 (S4_198565168) bin 4.08. A large number of SNPs associated with the trait also colocalized (< 20 Mb) to this region. This SNP was in close proximity to QTLs identified in previous studies for specific leaf area^21^ and chlorophyll content^40^ in maize seedlings exposed to chilling temperatures. Further the gene model associated within this region also mapped to a gene *dek44* (defective kernel44), encoding a mitochondrial ribosomal protein associated with seed development^66,67^. The SNP identified on bin2.04 (S2_47606952) and bin 7.00 (S7_2582951) were in close proximity to the SNPs identified for relative germination index, relative days to 50% root germination ^42^ and relative simple vitality index^43^ on a large panel of maize lines screened under low temperatures.

Cold stress is known to affect flowering machinery of maize leading to poor seed set^14^. As expected, in the current study cold stress delayed the flowering of the crop. However, this delay was not consistent across locations, and the flowering was delayed the most in L5 (Ludhiana_2019) followed by L4 (Varanasi_2019). These variations might be a result of possible differences in genetic mechanisms associated with delayed flowering in response to cold stress at different stages. The few co-localized SNPs identified for flowering across the environments is possibly a consequence of the profound variation observed in each of the environments. QTLs for flowering in maize have been reviewed extensively^68–71^with few of the loci also cloned or fine mapped^72,73^. In the current study, SNPs identified for male flowering across datasets were found on chromosome 3 (S3_186214261; bin3.06) and chromosome 9 (S9_90843159, bin9.03). In addition, the region on bin3.05–3.06 seems to be very important as a large number of SNPs co-localized to this bin. The gene models associated with the significant SNPs found with in this region were found to code for *S-formylglutathion hydrolase* and GDSL *esterase*/*lipase* (*LIP4*). GDSL esterase are largely involved in various plant developmental processes^74^, and play a major role in reproductive structures such as anther development and pollen dehiscensce in maize and Arabidopsis^75–78^. In addition, a cluster of SNPs (more than 7) were also identified for the across dataset on chromosome 2 (bin 2.03) and 8 (bin8.05) which were in close proximity (< 10 Mb) to identified genes for flowering *zfl2* and *vgt1*^73,79^. The gene model associated with the S9_90843159 (GRMZM2G056093) was associated with Tetratricopeptide repeat (TPR)-like superfamily proteins. Tetratricopeptide repeats are known to mediate protein interactions with partner proteins and are involved in plant stress and hormone signalling. Role of few TPRs during temperature stress for mediating removal of damaged proteins in Arabidopsis and maintaining homeostasis is well studied^80,81^. Two of the SNPs identified for female flowering on chromosomes 4 (S4_226942041) and 10 (S10_145198108) were near the QTNs identified in a previous study^33^ for female flowering in maize under cold stress. The SNP on chromosome 10 mapped to a region close to a flowering gene *zfl1*^82^.

Plant height (PH) is also one of the major selection factors in any breeding program. In the current study, reduction in plant height have been reported under cold stress across most of the locations. Several QTLs for plant height have been identified and genes for this trait cloned in maize^83^. Numerous SNPs for plant height across vegetative and flowering locations co-located in a region on chromosome 3 (bin 3.05-bin3.07), followed by SNPs on chromosome 1 (bin 1.01; 1.03–1.04) chromosome 2 (bin2.08) and chromosome5 (bin5.05). The region on chromosome 3 (S3_153769448) is known to harbour recessive semi-dwarf gene (*sdw2*)^84^. Incidentally this SNP also accounted for a large proportion (> 60%) of the phenotypic variation for the trait under vegetative stage cold stress tolerance. Most of the identified SNPs for plant height collocated to previously identified significant QTNs^11,85^.

Grain yield under cold stress being the primary trait was also subjected to GWAS in the current study and as expected, several significant SNPs were identified for this trait across locations. However, the study did not find any congruent SNPs across the four test locations revealing the complexity of the trait. The most significant SNPs for flowering stage cold stress tolerance dataset were identified on chromosomes1 (bin 1.11), 2 (bin2.08), 3 (bin3.05) and 7 (bin7.04) and on chromosomes 2 (bin2.02 &2.04) and 3 (bin3.06) for vegetative stage cold stress tolerance. The SNPs on chromosome 2 (bin 2.04 S2_45988012 and 2.08 S2_213163019) contributed substantially to the phenotypic variation under vegetative and flowering stage cold stress. As several of the significant SNPs were in LD, a haplotype trend regression was performed on the across yield dataset. The haplotype regression on grain yield revealed 6 haplotypes with a Bonferroni threshold (*P* value ≤ 0.05) on chromosome 2, 5, 6 and 9. The haplotypes identified on chromosome5 (bin5.05 and 5.07) had the lowest FDR. In addition, several SNPs associated with flowering and grain yield traits at most of the test location also collocated to bin 3.05. The gene models for most of these significant SNPs in this region were associated to enzymes such as *glutathione transferases*, *S-formylglutathione hydrolase* suggesting the significance of this region for cold stress tolerance in maize.

It has been widely understood that the cold stress adaptation in maize is a result of changes or modifications in cell structure, photosynthesis particularly PSII accompanied by changes in the development of maize and genes associated with these changes may contribute to cold stress tolerance. In the current study, the gene models for significant SNPs in the identified haplotypes for grain yield were associated with pentacotricopeptide repeat 60, ABC transporter protein, CID domain-containing protein, Zinc finger CCCH domain-containing protein 40. Several of these enzymes are associated with abiotic stress tolerance and/ or of cell wall constituents rendering them to be important in abiotic stress breeding. For instance, ABC transporter proteins are involved in maintaining cellular osmotic homeostasis and has been previously reported to contribute to tolerance to various abiotic stresses^86^. CID domain-containing protein and CCCH zinc-finger proteins regulate the adaptation of plants to abiotic stress. CCCH zinc-finger proteins improve the cold tolerance of plants by directly regulating the expression of downstream cold-related genes as transcription factors^87^. This further marks the importance of these regions in enhancing cold stress tolerance in maize.

The study also identified few hot spots where in significant SNPs for multiple traits for cold stress colocalized. The gene models associated with the significant SNPs within these regions have been previously associated with cold stress tolerance, such as serine/threonine *kinaes*, *S-formyl glutathione hydrolase* (SFGH), Calcium uniporter protein, Peptide transporter PTR2, kinesin-like protein KIN-4C, potassium channel proteins and transcription factors such as WRKY DNA-binding proteins. Several of these enzymes are associated with abiotic stress tolerance and/ or of cell wall constituents rendering them to be important in abiotic stress breeding. Families of *Serine/Threonine protein kinases* (*SNRK-2*) are known to enhance cold stress tolerance in Arabidopsis^88^. In addition, enhanced expression of few of *SNRK-2* genes have also been reported under cold stress in maize^89,90^. Further, WRKY transcription factors constitute one of the largest transcription factor families in plants and play important role in plant growth and thermal stress response^91^. As an outcome of this study, SNP assays are being developed for the SNPs identified in these hotspots, and the gene models associated with the most significant SNPs for secondary traits to validate them in breeding populations for them to be used for selections in the breeding pipeline.

## Conclusion

The study identified lines from the tropical maize breeding germplasm that are suitable for initiating cold stress tolerance breeding for developing and deploying hybrids directed at winter season cultivation of maize in South Asia. In addition, a suite of significant SNPs for grain yield and other secondary traits under cold stress in tropical maize germplasm were also identified in the study that could be of utility in these breeding pipelines. Several of these SNPs identified co-localized to QTLs previously reported for chilling tolerance, signifying their direct applicability in breeding programs post validation in independent breeding population. Further, genomic regions with cluster of SNPs for multiple cold related traits has also been identified particularly on chromosomes 1 (bin 1.01 &1.05), 4 (bin 4.05), 7 (bin 7.02) and chromosome8 (bin8.03) in this study, that would be of interest to further investigate. It was also observed that many of the SNPs identified explained minor effects suggesting the possibility of following a genomic selection approach, particularly in the initial stages of the breeding pipeline for enhanced and faster genetic gains.

## Materials and methods

An association mapping panel was constituted involving 297 diverse maize inbred lines sourced from CIMMYT’s lowland tropical maize program. The entries were a subset of a large panel (424) of working germplasm^92^ that have been used in previous studies used for identifying genomic regions for various abiotic stresses. The elite lines active in the breeding program and constituting the working germplasm of CIMMYT-Asia program involving both tropical and subtropical lineage. This panel of 297 lines were test-crossed with a CIMMYT tester line with high general combining ability (CML451) and phenotyped under natural field screens for cold stress associated traits under seedling, vegetative and flowering stage over five locations between the years 2018 and 2020.

### Experimental design

The test-cross progenies of association mapping panel involving 297 lines were planted using an alpha-lattice design with two replications across the four locations during the winter seasons of 2018 to 2020 across different target population of environments in India. The locations and planting dates within each locations were selected such that the lowest temperature period of the season coincided with the targeted crop growth stage viz., germination, vegetative and flowering stage (Table1). Each entry at the locations were planted in a 4-m long plot with a plant-to-plant spacing of 20 cm and a row-to-row spacing of 70 cm. Nitrogen (N) in the form of urea (60 kg ha^−1^) , Phosphorus ha^−1^ as single super phosphate (60 kg ha^−1^) and Potassium as muriate of potash (40 kg ha^−1^) along with 10 kg Zinc as zinc sulfate were applied as basal dose prior to planting. The second and third doses of N (each 30 kg ha^−1^) were side-dressed when plants were at knee-high and at tasselling stage, respectively. Pre-emergence application of pendimethalin and atrazine [both at 0.75 kg ha^−1^ active ingredient (a.i.), tank mixed] were used to keep the crop weed-free at early growth stages. Standard agronomic and plant protection measures were followed throughout the cropping period to prevent other biotic or abiotic stress affecting the trials.

### Phenotyping

The complete set of replicated trials involving the 297 entries were planted during the winter season at 5 locations, with planting dates adjusted such that the minimum temperature (< 15 °C) coincided with the critical growth stages of the crop representing the off-season environmental conditions that are prevalent in these TPEs. The different planting dates at each of the location and the targeted crop stage in these locations are detailed in the Table 1. The weather parameters during the crop growth stage at these locations are presented in Supplementary material Fig. 1. Observations were recorded on a plot basis for major agronomic traits such as days to 50 percent male and female flowering, plant height and plot yield. The complete plots of the trials were harvested and shelled, and the total kernel weight along with the moisture percentage was recorded. The grain yields were then converted to per hectare adjusted at 12.5 percent moisture content.

### Environmental variation

The trials were planted during the winter season at four locations (Table 1). Planting dates at each of these locations varied such that the crop could be exposed to low temperatures at varied growth stages (Table 1). The trial at location 1 (Ludhiana 2018) was planted during the second fortnight of December when the minimum daily temperatures reached below 4 °C and maximum daily temperature was < 20 °C (Supplimentary Fig. 1). The minimum temperatures (night) improved after a month but was still below < 10 °C. This trial location was used for studying the effect of low temperature on seedling germination and thus the plant stand. Two more trials were planted during October of the year 2018 at Locations 2 (L2: Samastipur 2018) & 3 (L3: Varanasi 2018) such that the pre flowering stage (V7 to VT) coincided with low temperatures. In both the locations, the temperatures during the first month of the planting and active vegetative growth stage were optimal (Tmax ~ 30 °C and Tmin ~ 15 °C). These trials experienced very low temperatures during the month of December and January (pre- flowering to flowering stage), when daily Tmin reached below 10 °C. The temperatures started to increase during the month of February. The trials in the second year of testing at locations 4 (L4: Ludhiana_2019) and 5 (L5: Varanasi_2019) were planted during November, such that the active vegetative stage of the trials coincided with low temperatures. As expected, the temperatures during the initial month of the planting were optimal (Tmax ~ 25 °C and Tmin ~  > 10 °C) and started to drop (Tmax < 20 °C and Tmin < 6 °C) from the second fortnight of December in both the locations. The sub-optimal minimum temperature (< 10 °C) regime continued for more than 3 months in location 4 (L4: Ludhiana_2019) extending the vegetative state of the crop.

### Genotyping

The panel of lines studied were genotyped using the GBS platform at the Institute for Genomic Diversity, Cornell University, Ithaca, NY, USA^93^. A total of 955,690 SNPs were generated through GBS v2.7 platform that were further filtered for the analysis, based on the method described by Suwarno et al.^94^ with slight modifications. A call rate of > 0.85 and minor allelic frequency (MAF) > 0.05 criteria resulted in a set of 213,273 SNPs for association mapping analysis. Marker density for association mapping panel was one SNP per 4.27 kb. Structure of the association panel was determined using principal components, wherein the SNPs were further filtered based on a call rate criterion of 0.9 and MAF < 0.1. In addition, Linkage Disequilibrium (LD) pruning based on adjacent markers was also carried out resulting in a set of 63,151 SNPs for principal component analysis.

### Statistical analysis

A linear mixed model was used for the analysis of the traits using the web handle META-R developed by CIMMYT^95^. The model for the analysis is represented as

$${\mathrm{Y}}_{\mathrm{ijkl}}=\upmu +{\mathrm{Env}}_{\mathrm{i}}+{\mathrm{Rep}}_{\mathrm{j}}({\mathrm{Env}}_{\mathrm{i}})+{\mathrm{Block}}_{\mathrm{k}}({\mathrm{Env}}_{\mathrm{i}}{\mathrm{Rep}}_{\mathrm{j}})+{\mathrm{Gen}}_{\mathrm{l}}+{\mathrm{Env}}_{\mathrm{i}}\times {\mathrm{Gen}}_{\mathrm{l}}+{\upvarepsilon }_{\mathrm{ijkl}}$$

Considering the strong relationship between grain yield and plant height, a covariate analysis was followed to obtain adjusted grain yield following the model

$${\mathrm{Y}}_{\mathrm{ijkl}}=\upmu +{\mathrm{Env}}_{\mathrm{i}}+{\mathrm{Rep}}_{\mathrm{j}}({\mathrm{Env}}_{\mathrm{i}})+{\mathrm{Block}}_{\mathrm{k}}({\mathrm{Env}}_{\mathrm{i}}{\mathrm{Rep}}_{\mathrm{j}})+{\mathrm{Gen}}_{\mathrm{l}}+{\mathrm{Env}}_{\mathrm{i}}\times {\mathrm{Gen}}_{\mathrm{l}}+{\mathrm{Cov}+\upvarepsilon }_{\mathrm{ijkl}}$$

where, Y_ijkl_ are the trait of interest, µ is the mean effect, Env_*i*_ is the effect of the i^th^ environment Rep_i_ is the effect of the i^th^ replicate, Block_j_ (Env_i_Rep_i_) is the effect of the j^th^ incomplete block within the i^th^ replicate and environment, Gen_l_ is the effect of the lth genotype, Env_i_ and Env_i_ × Gen_l_ are the effects of the i^th^ environment and the environment by genotype interaction, Cov is the effect of plant height and ε is the residual. All the effects were considered random except plant height which is considered fixed in the model.

The broad-sense heritability (h^2^) for individual and pooled dataset for the traits were estimated as ${\upomega }^{2}= {\upsigma }^{2}\mathrm{g }/({\upsigma }^{2}\mathrm{g }+{(\upsigma }^{2}\mathrm{e }/\mathrm{ R}))$ and ${\mathrm{h}}^{2}= {\upsigma }^{2}\mathrm{g }/({\upsigma }^{2}\mathrm{g }+{\upsigma }^{2}\mathrm{ge }/\mathrm{E }+ {\upsigma }^{2}\mathrm{e }/(\mathrm{E }\times \mathrm{ R}))$ respectively. Wherein, σ^2^g, σ^2^ge and σ^2^e represents variance components of genotype, genotype × environment and residuals, and number environments and replicates are represented as E and R.

Further to assess the stability of the genotypes across location, the grain yield from each for the entries tested were used to estimate coefficient of stability following Eberhart and Russell^96^. The genotypes with coefficient of regression close to 1 and low standard deviation were considered as adaptable and stable. The analysis was performed on CIMMYT data handle GEA-R^97^.

#### Association mapping

The analysis was carried out using multiple multi-locus models (Farm CPU, mrMLM, MLM, MLMM and BLINK) in GAPIT^98–102^. Association tests in between markers and association mapping panel phenotypes were corrected for population structure and kinship. The kinship matrix used as covariates were treated as random effects. The Quantile–quantile plots plotted using the observed − log 10P values and the expected − log 10P values for the primary trait of the study grain yield across the vegetative and cold stress environments were used to select the best model (BLINK)^102^ for association analysis based on observed minimal genomic inflation. A threshold of *p* value ≤ 1 × 10^–5^ was considered as a threshold for declaring a significant association. The associated SNPs were considered co-localized if found in proximity (within 1 Mb) to previously reported SNP for the trait or to be in the same chromosomal bin (www.maizegdb.org). In addition, the gene models with the putative SNP associations were identified from maize GDB genome browser at http://www.maizegdb.org (B73 RefGen_V2) and information on their prospective protein codes were obtained from Uniprot genome browser at http://uniprot.org.

Haplotype trait regression approach was also followed for the trait grain yield across environments. SNPs with a *P* value ≤ 0.0001for association in GWAS for grain yield in all the test locations were selected for haplotype detection and trait regression. Expectation maximization (EM) algorithm with 50 EM iterations, EM convergence tolerance of 0.0001, and a frequency threshold of 0.01 was used to estimate the haplotype frequency and block detection in SVS version 8.6.0. A step wise regression with forward elimination process was followed between the identified haplotype blocks and the trait grain yield across the four test locations.

## Supplementary Information

Supplementary Information 1.

Supplementary Information 2.

Supplementary Information 3.

Supplementary Information 4.

Supplementary Information 5.

Supplementary Information 6.

Supplementary Information 7.

## Data Availability

The phenotypic and the genotypic datasets used in the trials are deposited and available on CIMMYT Dataverse web handle (https://hdl.handle.net/11529/10548826). Additional information is also presented in the Supplementary Tables S1–S2.
