# Supplementary figures and images for: Genomic-regions associated with cold stress tolerance in Asia-adapted tropical maize germplasm
Source: Sci Rep. 2023 Apr 18;13:6297. doi: 10.1038/s41598-023-33250-8 (PMC10113201; doi:10.1038/s41598-023-33250-8)

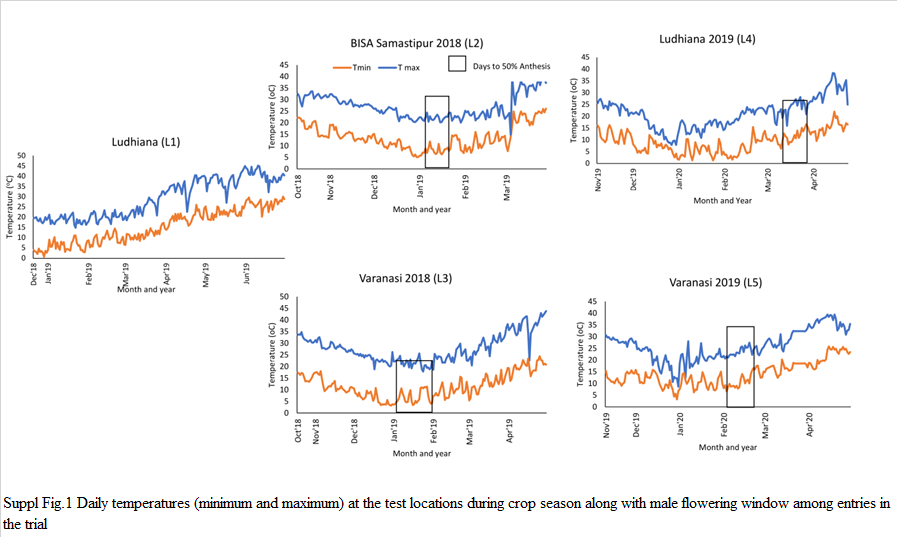

Supplement: Supplementary file 1 — Supplementary Information 1. [file 41598_2023_33250_MOESM1_ESM.jpg]

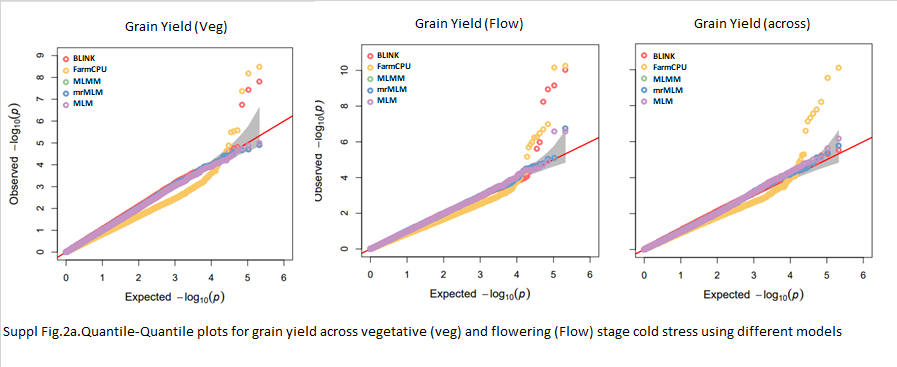

Supplement: Supplementary file 2 — Supplementary Information 2. [file 41598_2023_33250_MOESM2_ESM.jpg]

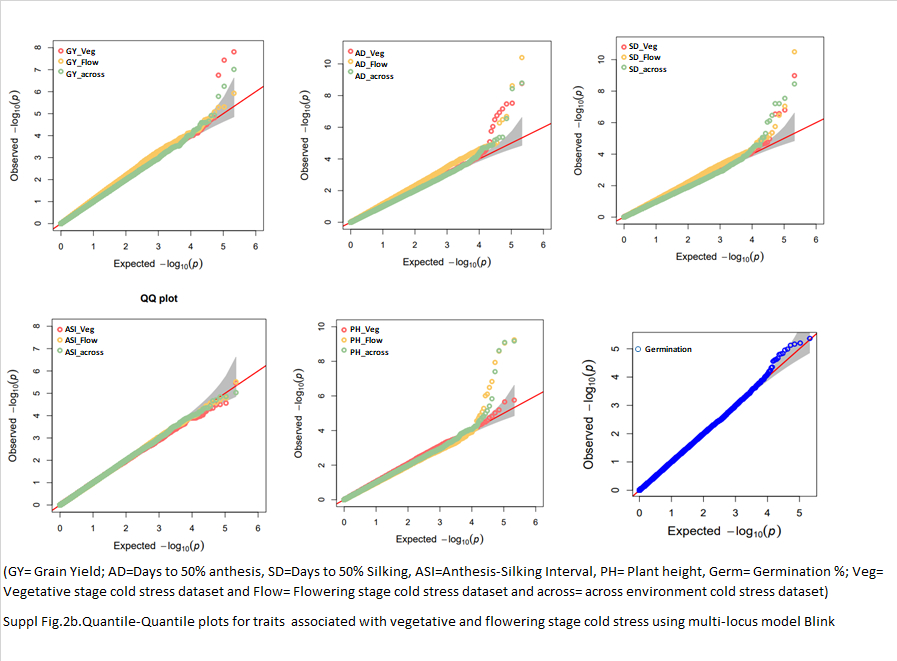

Supplement: Supplementary file 3 — Supplementary Information 3. [file 41598_2023_33250_MOESM3_ESM.jpg]

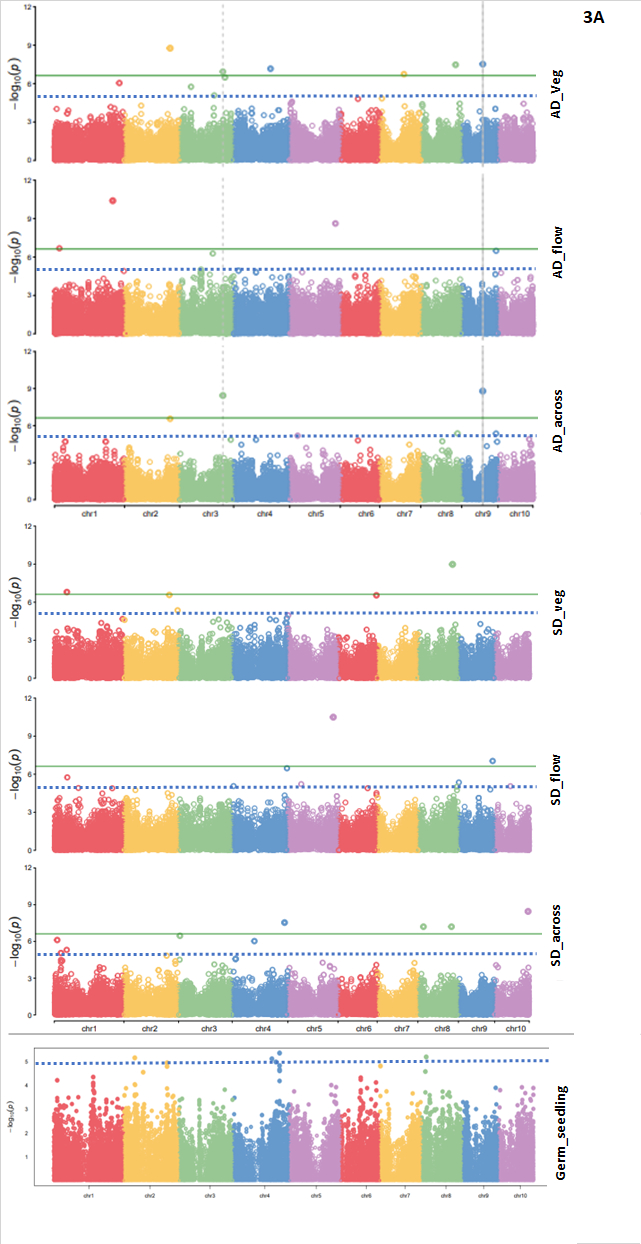

Supplement: Supplementary file 4 — Supplementary Information 4. [file 41598_2023_33250_MOESM4_ESM.jpg]

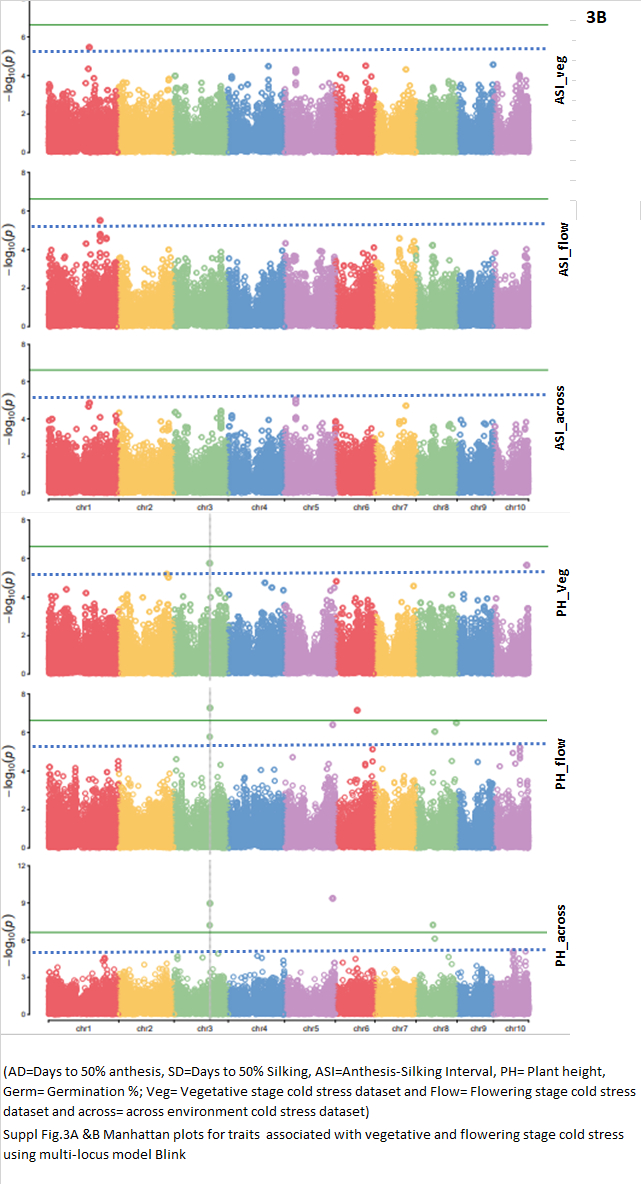

Supplement: Supplementary file 5 — Supplementary Information 5. [file 41598_2023_33250_MOESM5_ESM.jpg]
